# SIRT1 Activation by Resveratrol Alleviates Cardiac Dysfunction via Mitochondrial Regulation in Diabetic Cardiomyopathy Mice

**DOI:** 10.1155/2017/4602715

**Published:** 2017-08-13

**Authors:** Sai Ma, Jing Feng, Ran Zhang, Jiangwei Chen, Dong Han, Xiang Li, Bo Yang, Xiujuan Li, Miaomiao Fan, Congye Li, Zuhong Tian, Yabin Wang, Feng Cao

**Affiliations:** ^1^Department of Cardiology, Xijing Hospital, Fourth Military Medical University, Xi'an, Shaanxi, China; ^2^Department of Cardiology, Chinese PLA General Hospital, Beijing, China; ^3^Department of Emergency Medicine, Jinling Hospital, Nanjing, Jiangsu, China; ^4^Department of Digestive Diseases, Xijing Hospital, Fourth Military Medical University, Xi'an, Shaanxi, China

## Abstract

**Background:**

Diabetic cardiomyopathy (DCM) is a major threat for diabetic patients. Silent information regulator 1 (SIRT1) has a regulatory effect on mitochondrial dynamics, which is associated with DCM pathological changes. Our study aims to investigate whether resveratrol, a SRIT1 activator, could exert a protective effect against DCM.

**Methods and Results:**

Cardiac-specific SIRT1 knockout (SIRT1^KO^) mice were generated using Cre-loxP system. SIRT1^KO^ mice displayed symptoms of DCM, including cardiac hypertrophy and dysfunction, insulin resistance, and abnormal glucose metabolism. DCM and SIRT1^KO^ hearts showed impaired mitochondrial biogenesis and function, while SIRT1 activation by resveratrol reversed this in DCM mice. High glucose caused increased apoptosis, impaired mitochondrial biogenesis, and function in cardiomyocytes, which was alleviated by resveratrol. SIRT1 deletion by both SIRT1^KO^ and shRNA abolished the beneficial effects of resveratrol. Furthermore, the function of SIRT1 is mediated via the deacetylation effect on peroxisome proliferator-activated receptor gamma coactivator 1-alpha (PGC-1α), thus inducing increased expression of nuclear respiratory factor 1 (NRF-1), NRF-2, estrogen-related receptor-α (ERR-α), and mitochondrial transcription factor A (TFAM).

**Conclusions:**

Cardiac deletion of SIRT1 caused phenotypes resembling DCM. Activation of SIRT1 by resveratrol ameliorated cardiac injuries in DCM through PGC-1α-mediated mitochondrial regulation. Collectively, SIRT1 may serve as a potential therapeutic target for DCM.

## 1. Introduction

Diabetes mellitus is an emerging threat to global human health. According to an official report, diabetes will affect approximately 400 million patients universally [1]. Diabetic cardiomyopathy (DCM) has been a major cause for increased morbidity and mortality in diabetic patients, contributing to over 50% diabetic death [2]. Epidemiological studies have revealed that diabetic people have a 2- to 5-fold increase of risk in developing heart failure compared with age-matched healthy subjects, indicating the necessity of DCM research [3, 4]. DCM is characterized by left ventricular hypertrophy, fetal gene reactivation, and lipid accumulation in cardiac cells together leading to contractile dysfunction in myocardium [5, 6]. The pathophysiology of DCM is complex and not clearly elucidated, including mitochondrial dysregulation, inflammation, disruption of intracellular transport of Ca^2+^, and myocardium fibrosis [7]. Elucidation of the mechanisms for DCM is essential for the development of effective treatment strategies. Of particular interest, mitochondrial dysfunction has recently been reported to be a major contributor to the development of DCM. Mitochondrial dysfunction occurs by several mechanisms, involving impaired cardiac insulin and glucose homeostasis, impaired cellular and altered cardiac substrate metabolism, oxidative stress, and mitochondrial uncoupling [8]. Additionally, abundant evidence indicated that impaired mitochondrial biogenesis contributed to cardiac dysfunction in diabetic hearts [9]. Emerging evidence manifested that mitochondrial alterations might be a central mediator for the pathologic process in DCM. Therefore, searching for appropriate therapeutic approaches targeting mitochondrial biology holds a promise for the management of DCM [10].

Sirtuin 1 (SIRT1) is one of the seven mammalian homologs (SIRT1–SIRT7) of yeast silent information regulator 2 (Sir2). SIRT1 is an NAD^+^-dependent protein deacetylase [11]. It played multiple roles in cells including longevity, apoptosis, DNA repair, inflammation, and mitochondrial regulation [12]. As a pivotal protein in cellular metabolism, the regulatory effect of SIRT1 on mitochondrial dynamics has gained much attention. Recently, several studies reported that SIRT1 may play a beneficial role in DCM [13, 14], but the underlying mechanisms are not clearly elucidated.

In a previous study, 21 different molecules were identified as activators of SIRT1, of which resveratrol (2,3,4′-trihydroxystilbene) gained most attention [15]. Resveratrol, found to be linked to the cardiovascular benefits of red wine, has been shown to significantly increase SIRT1 activity through allosteric interaction, resulting in the increase of SIRT1 affinity for both NAD^+^ and the acetylated substrate [15, 16]. Resveratrol is a potential candidate for the treatment of cardiovascular diseases (including atherosclerosis, hypertension, myocardial ischemia, and heart failure), owing to its protective antioxidant, anti-inflammatory, and anti-angiogenic properties [17, 18]. In the majority of studies to date, resveratrol has been employed as an effective activator for SIRT1. In the work of Yu et al., Cote et al., and Liu et al., resveratrol was demonstrated to produce beneficial effects by enhancing the activation of SIRT1 [19–22].

To date, it is still unknown whether the regulatory effect of SIRT1 on mitochondrial dynamics could be beneficial in the pathological process of DCM. In the present study, we hypothesized that SIRT1 may exert a protective effect against the development of DCM through mitochondrial regulation. We applied DCM mouse model and *in vitro* high glucose (HG) cultured H9c2 cell model to investigate whether SIRT1 played an essential role in the development of DCM. To further confirm the crucial benefits of SIRT1, cardiac-specific SIRT1 knockout mice were generated and lentiviral vector targeting SIRT1 shRNA was synthesized. Besides, we investigated mitochondrial biogenesis and function indexes including mitochondrial DNA amount, ATP production, mitochondrial membrane potential, and mitochondrial morphological alterations. Finally, expressions of downstream proteins including PGC-1*α* were tested to determine the signaling pathway.

## 2. Methods

### 2.1. Ethics

All animal study procedures were performed in accordance with the Chinese National Institutes of Health. The experimental protocol was approved by the Fourth Military Medical University Committee on Animal Care.

### 2.2. Generation of the Cardiac-Specific SIRT1 Knockout (SIRT1^KO^) Mice

Cardiac-specific SIRT1 knockout mice (SIRT1^KO^) were generated by crossbreeding SIRT1^flox5-6/flox5-6^ with *Myh6*-Cre^+^ transgenic mice. SIRT1^flox5-6/flox5-6^ 129/FVB/Black/Swiss transgenic mice were generously presented by Professor Yongzhan Nie (State Key Laboratory of Cancer Biology and Xijing Hospital of Digestive Diseases, Xi'an, Shaanxi, China). Myh6-Cre C57BL/6a transgenic mice were purchased from the Jackson Lab (011038, the Jackson Laboratory, USA). Successful knockout of SIRT1 in myocardium was confirmed by PCR and Western Blot analysis. The PCR cycling conditions for *SIRT1* were a primary denaturation at 94°C for 5 min, followed by 30 cycles of 45 s at 94°C, annealing temperature at 59°C for 45 s, and 72°C for 45 s, with a final extension of 5 min at 72°C. Primer sequences for PCR were as follows: *Myh6*-Cre: forward *ATGACAGACAGATCCCTCCTATCTCC* and reverse *CTCATCACTCGTTGCATCATCGAC* and floxed *SIRT1* gene: forward *GTGGAGGTCAGAAGATCAACC* and reverse *CACATCTTACACAGATCCAC*.

### 2.3. Animal Grouping and Treatment

Mice were divided into six groups: control group (Con), diabetic cardiomyopathy group (DCM), DCM + resveratrol-treated group (DCM + RES), cardiac-specific SIRT1 knockout mouse group (SIRT1^KO^), SIRT1^KO^ + DCM group (SIRT1^KO^ + DCM), and SIRT1^KO^ + DCM + resveratrol-treated group (SIRT1^KO^ + DCM + R) (*n* = 10, each group). DCM mouse model was conducted as follows: eight-week-old mice were intraperitoneally injected with streptozotocin (STZ, Sigma, St. Louis, MO, USA) at the concentration of 150 mg/kg in citrate buffer (pH = 4.5) for seven consecutive days, while controlled mice received citrate buffer of the same volume. The blood glucose level was detected with a glucometer (Sannuo Biotech Ltd., Changsha, Hunan, China). *In vivo* experiments including echocardiography, PET/CT imaging, historical staining, and Western Blot were performed at least eight weeks after the establishment of a diabetic animal model. Mice with the fasting blood glucose level of higher than 350 mg/dL were considered as diabetic. RES-grouped mice were intraperitoneally treated with resveratrol of 25 mg/kg/d for five consecutive days. All animals had free access to water and food during the experiment. Animals were kept in plastic cages with well-ventilated stainless steel grid tops with a 12-hour light cycle (8am–8pm). The room temperature was maintained at 18–22°C.

### 2.4. Cell Culture and Treatment

High glucose (HG) culture conditions were used to mimic ex vivo DCM. H9c2 cardiomyoblast cell lines were used *in vitro* for underlying mechanisms. Cells were divided into six groups: control group (Con), high glucose group (HG), high glucose + resveratrol group (HG + R), lentiviral-transfected sh-SIRT1 group (SIRT1^KD^), sh-SIRT1 + high glucose group (SIRT1^KD^ + HG), sh-SIRT1 + high glucose + resveratrol group (SIRT1^KD^ + HG + R). H9c2 embryonic rat heart-derived cell line was purchased from the Shanghai Fuxiang Biotechnology Co. Ltd. (Shanghai, China) and cultured in DMEM medium (Hyclone, USA) containing 5 mmol/L D-glucose supplemented with 10% fetal bovine serum (FBS, Gibco, USA), 100 units/mL penicillin, and 100 mg/mL streptomycin. In the HG-treated group, cells were incubated with DMEM containing 30 mmol/L D-glucose. In the resveratrol-treated group, cells were cultured with additional 50 *μ*M resveratrol for 48 h. Cells were cultured at 37°C in a humidified atmosphere (95% air and 5% CO_2_).

### 2.5. Lentivirus Transfection

Recombinant lentiviral vectors that coexpressed green fluorescent protein (GFP) and SIRT1 shRNA were commercially constructed and purchased from GenePharma (Shanghai GenePharma Co., Ltd, Shanghai, China). Four different interfering sequences targeting SIRT1 were synthesized: number 1: 5′-*GCACCGATCCTCGAACAATTC*-3′, number 2: 5′-*GCAGGTTGCAGGAATCCAAAG*-3′, number 3: 5′-*GCCACCAACACCTCTTCATAT*-3′, and number 4: 5′-*GCCAGAGATTGTCTTCTTTGG*-3′. Optimal shRNA sequence with best interference efficiency was determined by real-time PCR and Western Blot. H9c2 cells were cultured in six-well plates and incubated with lentiviral sh-SIRT1 at different multiplicity of infections (MOI). Optimal MOI value was confirmed by GFP fluorescence imaging and flow cytometric method. After 24 h of transfection in complete medium, cells were cultured in HG or normal medium in shRNA-treated groups.

### 2.6. TMRM Fluorescence Imaging

Mitochondrial membrane potential was measured using tetramethylrhodamine methyl ester (TMRM) fluorescence imaging with a MitoPT TMRM Assay Kit (ImmunoChemistry Technologies, LLC, Bloomington, USA) according to the manufacturer's instruction. Fluorescence images were visualized by a confocal microscope (Olympus FV 1000, Olympus, Tokyo, Japan) at 543 nm excitation and 580 nm emission.

### 2.7. Mitochondrial Complex IV Enzyme Activity Assay

Mitochondrial complex IV enzyme activity was measured using a Complex IV Rodent Enzyme Activity Microplate Assay Kit (Abcam, USA) according to the manufacturer's instruction.

### 2.8. Quantitative Real-Time PCR

Total RNA was isolated from H9c2 cells or cardiac tissues. The cDNA was synthesized with the QuantiTect reverse transcription kit (Qiagen, Hiden, Germany). Real-time PCR was performed using the KAPA SYBR fast qPCR kit (KAPA Biosystems, Woburn, MA, USA). Primers of genes used in this experiment were synthesized commercially by TAKARA (TAKARA Biotechnology Co. Ltd., Dalian, Liaoning, China). The primer sequences are shown in Table 1. Amplification for genes of *NRF-1*, *NRF-2*, *ERR-α*, and *TFAM* was carried out with the initial incubation at 94°C for 30 s, followed by 40 cycles of the amplification step (94°C for 30 s, 60°C for 60 s, and 72°C for 1 min). Relative mRNA expressions were calculated by ΔΔCT method using the housekeeping gene GAPDH as an internal standard with 7500 System SDS Software version 1.2.1.22 (Applied Biosystems).

### 2.9. Mitochondrial DNA Amount

Mitochondrial DNA (mtDNA) amount was determined by the ratio of mtDNA to nucleic DNA, which were measured by quantitative real-time PCR. Quantitive PCR was performed using the following primer sequences: mtDNA-specific PCR: forward *CCGCAAGGGAAAGATGAAAGA* and reverse *TCGTTTGGTTTCGGGGTTTC* and nuclear DNA-specific PCR: forward *GCCAGCCTCTCCTGATGT* and reverse *GGGAACACAAAAGACCTCTTCTGG*. The PCR amplification conditions were a primary denaturation at 94°C for 10 min, followed by 30 cycles of 1 min at 94°C, 1 min at 56°C, and 1 min at 72°C, with a final extension of 5 min at 72°C.

### 2.10. Western Blot Analysis

Myocardium tissue was harvested for Western blot as described previously [23]. Cells of each group were harvested at appropriate time. Cells were washed three times with PBS and collected after ice-cold lysis buffer digestion. Protein lysates were separated on 10% SDS-PAGE gels and transferred onto nitrocellulose (NC) membrane. Membranes were blocked with 5% milk in 1 × TBS-Tween-20 buffer and incubated overnight at 4°C with primary antibodies. Then, membranes were washed in Tris-buffered saline with Tween, followed by incubation with the corresponding secondary antibodies at room temperature for 1 h. The blots were developed using an enhanced chemiluminescence kit (Millipore, Billerica, MA, USA) and visualized with UVP Bio-Imaging Systems. Blot densities were analyzed using ImageJ Software (National Institutes of Health, Bethesda, MD).

Primary antibodies are the following: anti-SIRT1, anti-IRS2, anti-P-Akt S473, anti-P-Akt T308, anti-t-Akt, anti-acetylated protein, anti-PGC-1*α*, anti-NRF1, anti-NRF2, anti-ERR-*α*, anti-TFAM, anti-GAPDH, and anti-*β*-actin (all from Abcam, Cambridge, MA, USA). Secondary antibodies are the following: horseradish peroxidase-conjugated goat anti-rabbit and goat anti-rat (from Zhongshan Biotechnology Co. Ltd., Beijing, China).

### 2.11. TUNEL Assay

TUNEL assay was conducted using a commercial Cell Death Detection Kit (Roche, Penzberg, Germany) according to the manufacturer's instructions. Images were obtained with a fluorescence microscope (Olympus, Shinjuku, Tokyo, Japan). The index of apoptosis was expressed as the proportion of the TUNEL-positive cells to the total cells.

### 2.12. Historical Staining

Hearts were fixed in a 4% paraformaldehyde solution, embedded in paraffin, and sectioned at 5 mm. Sections were stained with hematoxylin-eosin (H-E) or Masson's trichrome staining. Images of sections were visualized using a light microscope (Olympus, Japan).

### 2.13. Cardiac Function Evaluation by Echocardiography

Mouse cardiac function was performed with Vevo 2100 ultrasound system (Visual-Sonics, Toronto, Canada) with a 30 MHz linear transducer. Anesthesia was conducted with inhaled 1.0% isoflurane in oxygen. Animals were placed on a warming pad during the whole process. Left ventricular end-diastolic diameter (LVEDd), left ventricular end-systolic diameter (LVESd), left ventricle (LV) internal dimension in diastole (LVID, d) and systole (LVID, s), and interventricle septem thickness in diastole (IVS, d) and systole (IVS, s) were measured. All measurements were based on 6 consecutive cardiac cycles. Left ventricular ejection fraction (LVEF) and fractional shortening (FS) were calculated by computer algorithms. All of these measurements were performed by a blinded investigator.

### 2.14. ^18^F-Fluorodeoxyglucose Positron Emission Tomography/Computed Tomography

^18^F-Fluorodeoxyglucose (^18^F-FDG) positron emission tomography (PET)/computed tomography (CT) scanning was performed using an animal PET/CT scanner (Mediso Nano PET/CT, Mediso Medical Imaging Systems, Budapest, Hungary) to evaluate cardiac glucose metabolism as described previously [24]. Briefly, mice were maintained under fasting condition for about 12 h and to keep glucose levels between 6.0 and 7.5 mmol/L. Animals were injected with 1 mCi of ^18^F-FDG via the tail vein 30 min before PET/CT imaging. CT scan (45 kV, 179 *μ*A) and PET scan were performed successively during 20 min and 30 min in two frames. Images were reconstructed using the ordered subset expectation maximization reconstruction algorithm with decay, attenuation, and random correction form raw framed sinograms. Processed images were displayed in a sagittal plane.

### 2.15. Transmission Electron Microscopy (TEM)

TEM was performed to observe morphological mitochondrion changes in myocardium as previously described. Briefly, hearts were removed from anesthetized mice and washed with PBS solution. A specimen of the left ventricular myocardium was cut into ultrathin sections with a thickness of 60–64 nm. Sections were taken after fixation, stepwise alcohol dehydration, embedding, polymerization, sectioning, and staining. Images were observed with an electron microscope (JEM-2000EX TEM, JEOL Ltd., Tokyo, Japan). Random sections were taken and visualized by a blinded technician.

### 2.16. Statistical Analysis

Data was expressed as mean ± standard deviation (SD). SPSS15.0 (SPSS Inc., USA) and Prism5.0 (GraphPad Software, USA) were used to perform the one-way analysis of variance (ANOVA) for evaluating the differences among different experimental groups. Pairwise multiple comparisons were used to identify the parameter differences between the two groups using the ANOVA-conjuncted Tukey test. Data was analyzed using parametric test assuming Gaussian distribution. *p* value < 0.05 was considered significant.

## 3. Results

### 3.1. Resveratrol Alleviated Cardiac Dysfunction in DCM Mouse Heart

**Figure 1:**
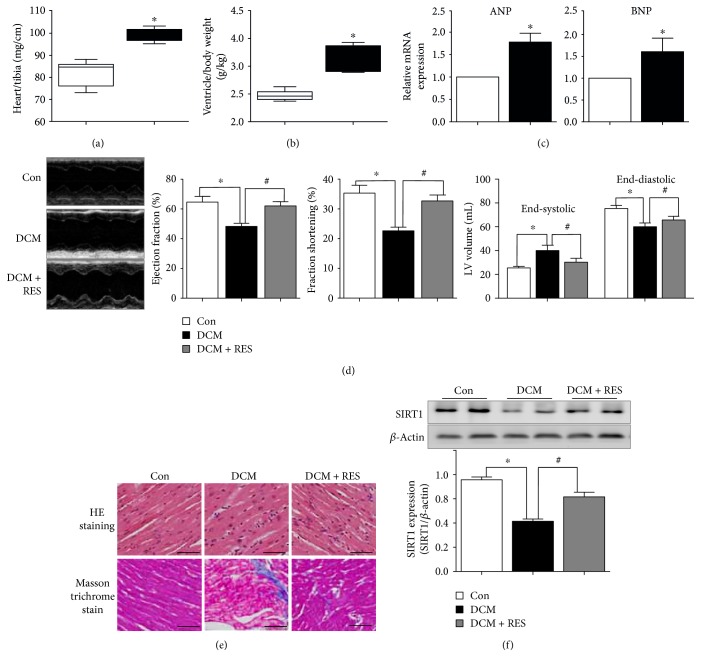
Resveratrol alleviated cardiac dysfunction in DCM mouse heart. (a) Increased heart weight/tibia length in STZ-induced DCM mice (^∗^*p* < 0.05). (b) Increased ventricle/body weight in DCM mice as compared with Con mice (^∗^*p* < 0.05). (c) Elevated expression of myocardial hypertrophy markers of atrial natriuretic peptide (ANP) and brain natriuretic peptide (BNP) in DCM mouse heart (^∗^*p* < 0.05). (d) Impaired cardiac function in diabetic hearts (^∗^*p* < 0.05), while resveratrol treatment significantly reversed these changes in DCM mice (^#^*p* < 0.05). (e) DCM mouse hearts displayed structural changes and fibrogenesis, and resveratrol markedly alleviated these changes. (f) SIRT1 was markedly downregulated in DCM mouse myocardium (^∗^*p* < 0.05), while resveratrol enhanced SIRT1 expression in DCM + RES mice (^#^*p* < 0.05). ^∗^*p* < 0.05 versus Con; ^#^*p* < 0.05 versus DCM.

Ventricular hypertrophy, myocardial fibrosis, and cardiac dysfunction are major characteristics of DCM hearts. As shown in Figures 1(a) and 1(b), myocardial hypertrophy in STZ-induced DCM mice was characterized by increased heart weight/tibia length (89.2 ± 2.86 versus 72.0 ± 5.73, *p* < 0.05, DCM versus Con group) and enhanced ventricle/body weight (g/kg, 3.16 ± 0.22 versus 2.47 ± 0.09, *p* < 0.05, DCM versus Con group). In addition, myocardial hypertrophy in DCM was also evidenced by increased expression of atrial natriuretic peptide (ANP) and brain natriuretic peptide (BNP) (Figure 1(c)). Echocardiography results (Figure 1(d)) showed that cardiac functions, namely, ejection fraction (EF) and fractional shortening (FS), were pointedly reduced in DCM mice (EF: 48.3 ± 3.51% versus 64.7 ± 6.51%, *p* < 0.05, DCM versus Con group; FS: 22.7 ± 2.08% versus 35.3 ± 4.51%, *p* < 0.05, DCM versus Con group). Comparing with DCM mice, resveratrol treatment significantly elevated EF and FS in DCM + RES mouse heart (EF: 62.0 ± 5.00% versus 48.3 ± 3.51%, *p* < 0.05, DCM + RES versus DCM group; FS: 32.7 ± 3.51% versus 22.7 ± 2.08%, *p* < 0.05, DCM + RES versus DCM group). Moreover, both end-systolic and end-distolic volumes were increased in DCM mice as compared with Con mice (end-systolic volume: 40.1 ± 4.46 versus 25.5 ± 1.33, *p* < .05, DCM versus Con group; end-distolic volume: 60.0 ± 3.20 versus 75.4 ± 2.54, *p* < 0.05, DCM versus Con group), while resveratrol significantly reduced end-systolic and end-distolic volumes in DCM + RES mice (end-systolic volume: 30.3 ± 3.29 versus 40.1 ± 4.46, *p* < 0.05, DCM + RES versus DCM group; end-distolic volume: 65.7 ± 3.03 versus 60.0 ± 3.20, *p* < 0.05, DCM + RES versus DCM group). Comparing to the Con group, DCM mouse hearts displayed structural changes, including unbalanced cellular structures, broken fibers, and the aggregation of necrotic and inflammatory myocytes (HE staining, Figure 1(e)). Additionally, masson trichrome staining demonstrated bits of fibrogenesis in the myocardium of the DCM group, and resveratrol markedly alleviated these changes in DCM mice (Figure 1(e)). We further examined SIRT1 expression in myocardium. Western Blot results demonstrated that SIRT1 was markedly downregulated in DCM mouse myocardium (0.42 ± 0.02 versus 0.76 ± 0.03, *p* < 0.05, DCM versus Con group), while resveratrol enhanced SIRT1 expression in DCM + RES mice (0.62 ± 0.05 versus 0.42 ± 0.02, *p* < 0.05, DCM + RES versus DCM group), suggesting that the reduction of DCM cardiac function and beneficial effects of resveratrol may be associated with SIRT1.

### 3.2. Generation and Screening of Cardiac-Specific SIRT1 Knockout (SIRT1^KO^) Mice

**Figure 2:**
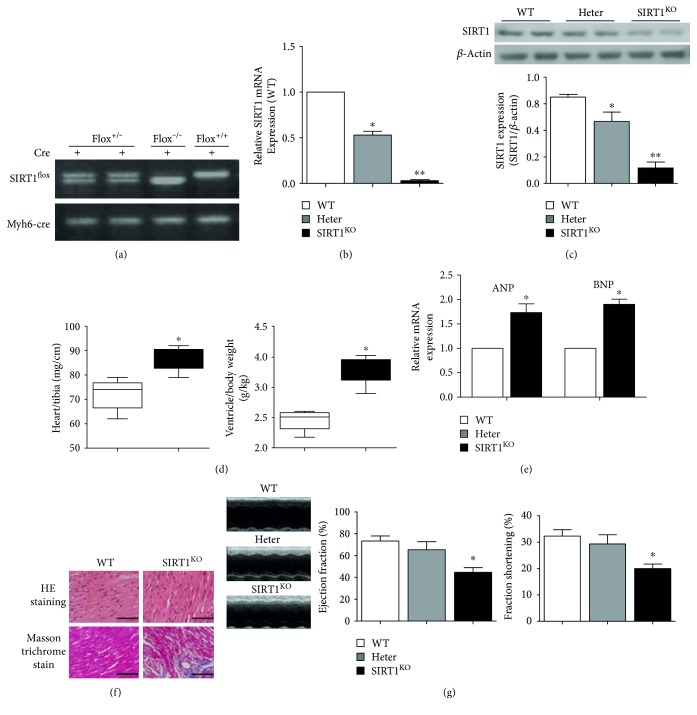
Cardiac-specific SIRT1 knockout (SIRT1^KO^) mice displayed symptoms of DCM. (a) Mouse tail tissue PCR showed different genotypes during the process of crossbreeding. (b) There was almost no SIRT1 mRNA in SIRT1^KO^ mouse cardiac tissue (^∗^*p* < 0.05 in Heter group, ^∗∗^*p* < 0.01 in SIRT1^KO^ group). (c) SIRT1 protein was also barely expressed in SIRT1^KO^ mice myocardium (^∗∗^*p* < 0.01 in SIRT1^KO^ group). (d) Increased heart weight/tibia length ratio in SIRT1^KO^ mouse myocardium and ventricle/heart weight in SIRT1^KO^ mouse myocardium (^∗^*p* < 0.05). (e) The mRNA expressions of atrial natriuretic peptide (ANP) and brain natriuretic peptide (BNP) were significantly increased in SIRT1^KO^ mice (^∗^*p* < 0.05). (f) SIRT1^KO^ mouse hearts displayed structural changes and fibrogenesis in the myocardium. (g) Cardiac function was impaired in SIRT1^KO^ mice as compared with WT and Heter mice (^∗^*p* < 0.05) (Figure 1(g)). ^∗^*p* < 0.05 versus WT. WT: wild type; Heter: heterozygous.

Cardiac-specific SIRT1 knockout (SIRT1^KO^) mice were obtained through the crossbreeding of SIRT1^flox5-6/flox5-6^ and *Myh6*-Cre transgenic mice. Mouse tail tissue PCR (Figure 2(a)) showed different genotypes during the process of crossbreeding. Mice with genotype of SIRT1^flox+/−^ and Myh6-Cre^+^ (shown in the first two columns in Figure 2(a)) were perceived as heterozygous (Heter) mice in which SIRT1 was partially expressed in myocardium. Mice with genotype of SIRT1^flox−/−^ and Myh6-Cre^+^ (same background, but normal SIRT1 expression, shown in the third column in Figure 2(a)) were used as control mice. Mice with genetype of SIRT1^flox+/+^ and Myh6-Cre^+^ (shown in the fourth column in Figure 2(a)) were considered as SIRT1^KO^ mice.  Figure 2(b) shows that there was almost no SIRT1 mRNA in SIRT1^KO^ mouse cardiac tissue (0.53 ± 0.07 in the Heter group, and 0.03 ± 0.02 in the SIRT1^KO^ group). In addition, SIRT1 protein was also barely expressed in SIRT1^KO^ mouse myocardium (Figure 2(c)), indicating the successful knockout of SIRT1 in myocardium. As for the organ-specific knockout characteristics of Cre-loxP recombination system, SIRT1 was normally expressed in other organs such as the lungs, kidneys, and brain (shown in Supplementary Figures S1A and S1B available online at https://doi.org/10.1155/2017/4602715).

### 3.3. SIRT1^KO^ Mice Displayed Symptoms of DCM

Similar to DCM hearts, hearts from SIR1^KO^ mice were enlarged with ventricular hypertrophy and cardiac dysfunction.  Figure 2(d) reveals an increased heart weight/tibia length ratio (86.5 ± 4.76 versus 72.2 ± 6.15, *p* < 0.05, SIR1^KO^ versus WT group) and ventricle/heart weight (g/kg, 2.95 ± 0.17 versus 2.34 ± 0.12, *p* < 0.05, SIR1^KO^ versus WT group) in SIRT1^KO^ mice as compared with WT mice. The mRNA expressions of atrial natriuretic peptide (ANP) and brain natriuretic peptide (BNP) were significantly increased in SIRT1^KO^ mice (*p* < 0.05, SIRT1^KO^ versus WT group) (Figure 2(e)). Resembling DCM mice, SIRT1^KO^ mouse hearts also displayed structural changes, such as unbalanced cellular structures or aggregation of necrotic and inflammatory myocytes, and masson trichrome staining demonstrated bits of fibrogenesis in the myocardium of SIRT1^KO^ heart (Figure 2(f)). More importantly, cardiac function indicators of EF and FS were significantly decreased in SIRT1^KO^ mice as compared with WT and Heter mice (EF: 44.7 ± 7.51% versus 73.3 ± 8.08%, *p* < 0.05, SIRT1^KO^ versus WT group; FS: 20.0 ± 3.00% versus 32.3 ± 4.16%, *p* < 0.05, SIRT1^KO^ versus WT group) (Figure 1(g)). These data suggest that cardiac-specific SIRT1 knockout of SIRT1 was sufficient to duplicate the phenotype of DCM, demonstrating that the existence of SIRT1 may exert a crucial role in cardiac function and structure modeling in diabetic hearts.

### 3.4. Myocardial Metabolic and Mitochondrial Alterations in DCM and SIRT1^KO^ Mice

**Figure 3:**
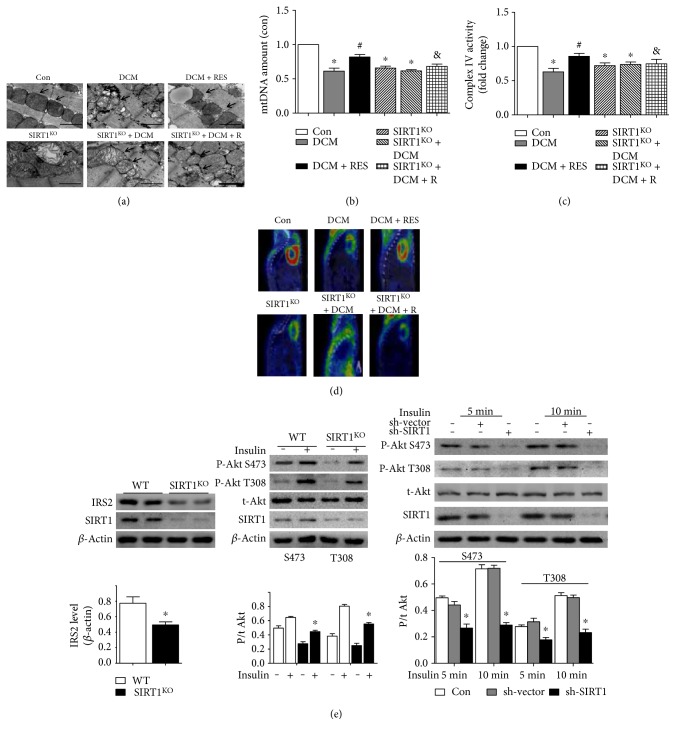
Myocardial metabolic and mitochondrial alterations in DCM and SIRT1^KO^ mice. (a) TEM images revealed morphological mitochondrial impairment in both DCM and SIRT1^KO^ mice, and SIRT1 activation by resveratrol could alleviate these mitochondrial changes in DCM + RES mice. (b) Mitochondrial DNA (mtDNA) amount was significantly decreased in the DCM, SIRT1^KO^, and SIRT1^KO^ + DCM groups as compared with the Con group (^∗^*p* < 0.05). Resveratrol elevated mtDNA amount in the DCM + R group (^#^*p* < 0.05), while resveratrol's protective effects were diminished in the SIRT1^KO^ + DCM + R group as compared with the DCM + RES group (^&^*p* < 0.05). (c) Complex IV activity was significantly reduced in the DCM, SIRT1^KO^, and SIRT1^KO^ + DCM groups (^∗^*p* < 0.05) than in the Con group. Resveratrol markedly alleviated complex IV activity reduction in DCM mice (^#^*p* < 0.05). While in contrast, the beneficial effect of resveratrol was completely abolished in the SIRT1^KO^ + DCM + R group (^&^*p* < 0.05, SIRT1^KO^ + DCM + R versus DCM + RES). (d) Both DCM and SIRT1^KO^ led to defective ^18^F-FDG uptake in myocardium, and resveratrol ameliorated this in DCM + R mice but not in SIRT1^KO^ + DCM + R. (e) Insulin receptor substrate 2 (IRS2) protein level was reduced to about 60% in SIRT1^KO^ mouse hearts relative to WT mice, and impaired insulin signaling was revealed by reduced myocardial Akt phosphorylation in response to insulin stimulation (^∗^*p* < 0.05). *In vitro* insulin-induced phosphorylation of Akt at both T308 and S473 sites was also significantly diminished by SIRT1 sh-RNA (^∗^*p* < 0.05). ^∗^*p* < 0.05 versus Con; ^#^*p* < 0.05 versus DCM; ^&^*p* < 0.05 versus DCM + RES.

TEM images (Figure 3(b)) revealed morphological and mitochondrial impairment in both DCM and SIRT1^KO^ mice. Control heart showed normal tightly packed interfibrillar mitochondrion appearance. While in DCM and SIRT1^KO^ myocardium, interfibrillar mitochondria were observed less uniform and more fragmented, displaying swollen appearance with loss of discernable cristae. Excessive accumulation of glucose granules was also observed in DCM heart. Additionally, SIRT1 activation by resveratrol could alleviate these mitochondrial changes in DCM + RES mice (Figure 3(a)). Mitochondrial DNA (mtDNA) amount was an effective indicator for mitochondrial biogenesis. Figure 3(b) demonstrates that mtDNA amount was significantly decreased in the DCM, SIRT1^KO^, and SIRT1^KO^ + DCM groups as compared with the Con group (DCM 0.61 ± 0.08, SIRT1^KO^ 0.66 ± 0.05, and SIRT1^KO^ + DCM 0.62 ± 0.03; *p* < 0.05), while resveratrol's protective effects were diminished in the SIRT1^KO^ + DCM + R group as compared with the DCM + RES group (0.68 ± 0.06 versus 0.82 ± 0.06, *p* < 0.05, SIRT1^KO^ + DCM + R versus DCM + RES). Complex IV activity (Figure 3(c)) manifested the changes in mitochondrial function. Complex IV activity was significantly reduced in the DCM, SIRT1^KO^, and SIRT1^KO^ + DCM groups (DCM 0.63 ± 0.09; SIRT1^KO^ 0.72 ± 0.07; and SIRT1^KO^ + DCM 0.73 ± 0.06, *p* < 0.05) than that in the Con group. Resveratrol markedly alleviated complex IV activity reduction in DCM mice (0.86 ± 0.08 versus 0.63 ± 0.09, *p* < 0.05, DCM + RES versus DCM). While in contrast, the beneficial effect of resveratrol was completely abolished in the SIRT1^KO^ + DCM + R group (0.77 ± 0.12 versus 0.86 ± 0.08, *p* < 0.05, SIRT1^KO^ + DCM + R versus DCM + RES). PET/CT scanning revealed metabolic glucose uptake changes in myocardium. It could be inferred that DCM led to defective ^18^F-FDG uptake in myocardium, which was significantly improved by resveratrol. Interestingly, SIRT1^KO^ also resulted in decreased ^18^F-FDG uptake, but resveratrol did not ameliorate this in SIRT1^KO^ + DCM + R mice (Figure 3(d)).

### 3.5. SIRT1 Downregulation in H9c2 Cells by shRNA Lentiviral Vector

GFP fluorescence images and flow cytometry results showed that H9c2 cells were optimally transfected at the MOI of 100 (Supplementary Figures S2A and S2B), which was the selected concentration in our later experiments. Western blot was done to reveal the effectiveness of SIRT1 downregulation in four different shRNA interfering sequences (Supplementary Figure S2C). As compared with other three sequences, number 1 sequence decreased SIRT1 expression to the largest extent (0.26 ± 0.05 versus 0.50 ± 0.04, *p* < 0.01, number 1 sequence versus Con) and was used in our later experiment in the SIRT1 knockdown (SIRT1^KD^) group. As compared to the Con group, HG treatment significantly reduced SIRT1 expression in H9c2 cells (0.65 ± 0.12 versus 0.85 ± 0.12, *p* < 0.05, HG versus Con), and resveratrol pointedly elevated SIRT1 in HG cells (0.83 ± 0.06 versus 0.64 ± 0.12, *p* < 0.05, HG + RES versus HG). Additionally, resveratrol did not reverse SIRT1 downregulation in SIRT1^KD^ cells due to the knockdown efficacy of shRNA (0.49 ± 0.06 versus 0.49 ± 0.03, *p* > 0.05, SIRT1^KD^ + HG + R versus SIRT1^KD^ + HG) (Supplementary Figure S2D).

### 3.6. SIRT1 Low Expression Led to Cardiac Insulin Resistance

Insulin signaling pathway is a critical pathway regulating cellular energy metabolism, and insulin resistance may be one of the reasons resulting in metabolic disturbance in SIRT1 low-expressed hearts. As was revealed by Western blot (Figure 3(e)), insulin receptor substrate 2 (IRS2) protein level was reduced to about 60% in SIRT1^KO^ mouse hearts relative to WT mice, which was expected to cause deterioration in insulin signaling. As expected, impaired insulin signaling was revealed by reduced myocardial Akt phosphorylation in response to insulin stimulation (P-Akt S473/total Akt: 0.45 ± 0.02 versus 0.65 ± 0.02, *p* < 0.05, SIRT1^KO^ versus WT; P-Akt T308/total Akt: 0.55 ± 0.02 versus 0.81 ± 0.03, *p* < 0.05, SIRT1^KO^ versus WT). We further investigated insulin signaling in an *in vitro* setting by shRNA lentiviral vector. As expected, insulin-induced phosphorylation of Akt at both T308 and S473 sites was significantly diminished by SIRT1 low expression (P-Akt S473/total Akt, 5 min: 0.27 ± 0.03 versus 0.50 ± 0.01, *p* < 0.05, sh-SIRT1 versus Con; P-Akt S473/total Akt, 10 min: 0.29 ± 0.02 versus 0.71 ± 0.03, *p* < 0.05, sh-SIRT1 versus Con; P-Akt T308/total Akt, 5 min: 0.18 ± 0.02 versus 0.28 ± 0.01, *p* < 0.05, sh-SIRT1 versus Con; and P-Akt T308/total Akt, 10 min: 0.23 ± 0.03 versus 0.51 ± 0.02, *p* < 0.05, sh-SIRT1 versus Con).

### 3.7. High Glucose and SIRT1^KD^ Impaired Mitochondrial Biogenesis and Function

**Figure 4:**
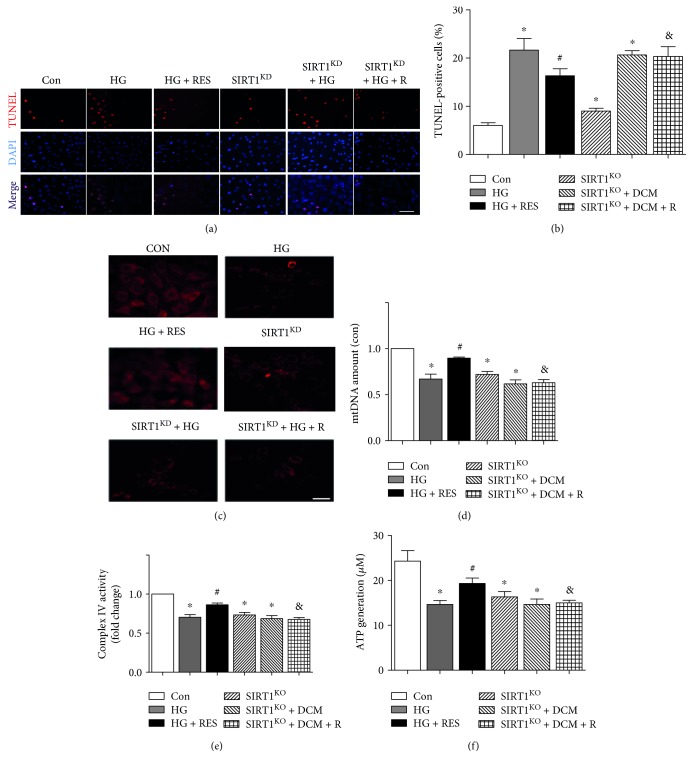
High glucose and SIRT1 deletion impaired mitochondrial biogenesis and function. (a and b) Both HG and SIRT1 deletion by shRNA lentiviral vector increased apoptotic cell ratio in H9c2 cells (^∗^*p* < 0.05), but resveratrol did not suppress HG-induced apoptosis in SIRT1^KD^ + HG + R cells as in HG + RES cells (^#^*p* < 0.05, ^&^*p* < 0.05). (c) Control cells showed strong TMRM fluorescence, while HG and SIRT1^KD^ resulted in decreased TMRM fluorescence due to depolarization of the mitochondria. Additionally, resveratrol relatively increased TMRM fluorescence in HG + RES cells but not in SIRT1^KD^ + HG + R cells. (d) mtDNA amount was reduced in HG and SIRT1^KD^-treated cells (^∗^*p* < 0.05), and resveratrol markedly increased mtDNA amount in the HG + RES group but not in the SIRT1^KD^ + HG + R group (^#^*p* < 0.05, ^&^*p* < 0.05). (e) Mitochondrial enzyme activity was reduced in HG and SIRT1^KD^-grouped cells (^∗^*p* < 0.05), and resveratrol markedly elevated it in the HG + RES group but not in the SIRT1^KD^ + HG + R group (^#^*p* < 0.05, ^&^*p* < 0.05). (f) ATP generation demonstrated the similar tendency with mitochondrial enzyme activity. ^∗^*p* < 0.05 versus Con; ^#^*p* < 0.05 versus DCM; ^&^*p* < 0.05 versus DCM + RES.

Both HG and SIRT1^KD^ by shRNA lentiviral vector increased apoptotic cell ratio in H9c2 cells (21.7 ± 4.16 versus 6.0 ± 1.00, *p* < 0.05, HG versus Con; 9.00 ± 1.00 versus 6.00 ± 1.00, *p* < 0.05, SIRT1^KD^ versus Con), but resveratrol did not suppress HG-induced apoptosis in SIRT1^KD^ + HG + R cells as in HG + RES cells (16.3 ± 2.52 versus 21.7 ± 4.16, *p* < 0.05, HG + R versus HG; 20.3 ± 3.51 versus 20.7 ± 1.53, *p* > 0.05, SIRT1^KD^ + HG + R versus SIRT1^KD^ + HG) (Figures 4(a) and 4(b)). Mitochondrial membrane potential (MMP) was measured using TMRM fluorescence imaging. Control cells showed strong TMRM fluorescence indicative of normal membrane potential, while HG and SIRT1^KD^ resulted in decreased TMRM fluorescence due to depolarization of the mitochondria. Additionally, resveratrol relatively increased TMRM fluorescence in HG + RES cells but not in SIRT1^KD^ + HG + R cells (Figure 4(c)). mtDNA amount indicating mitochondrial biogenesis was reduced in HG and SIRT1^KD^-treated cells (0.67 ± 0.09/Con, *p* < 0.05, HG versus Con; 0.72 ± 0.06/Con, *p* < 0.05, SIRT1^KD^ versus Con), and resveratrol markedly increased mtDNA amount in the HG + RES group (0.90 ± 0.02/Con, *p* < 0.05, HG + RES versus HG) (Figure 4(d)). Mitochondrial complex IV enzyme activity was measured to indicate mitochondrial function.  Figure 4(e) reveals that mitochondrial enzyme activity was reduced in HG and SIRT1^KD^-grouped cells (0.70 ± 0.06/Con, *p* < 0.05, HG versus Con; 0.73 ± 0.06/Con, *p* < 0.05, SIRT1^KD^ versus Con) and resveratrol markedly elevated it in the HG + RES group (0.86 ± 0.04/Con, *p* < 0.05, HG + RES versus HG) but not in the SIRT1^KD^ + HG + R group (0.68 ± 0.04 versus 0.69 ± 0.07, *p* > 0.05, SIRT1^KD^ + HG + R versus SIRT1^KD^ + HG). ATP generation was also a strong indicator for mitochondrial function, which demonstrated the similar tendency with mitochondrial enzyme activity (14.7 ± 1.53 versus 24.3 ± 4.08, *p* < 0.05, HG versus Con; 16.3 ± 2.08 versus 24.3 ± 4.08, *p* < 0.05, SIRT1^KD^ versus Con; 19.3 ± 2.08 versus 14.7 ± 1.53, *p* < 0.05, HG + RES versus HG; and 15.0 ± 1.00 versus 14.7 ± 2.08, *p* > 0.05, SIRT1^KD^ + HG + R versus SIRT1^KD^ + HG) (Figure 4(f)).

### 3.8. SIRT1 Regulated Mitochondrial-Related Protein Expression through PGC-1*α* Deacetylation

**Figure 5:**
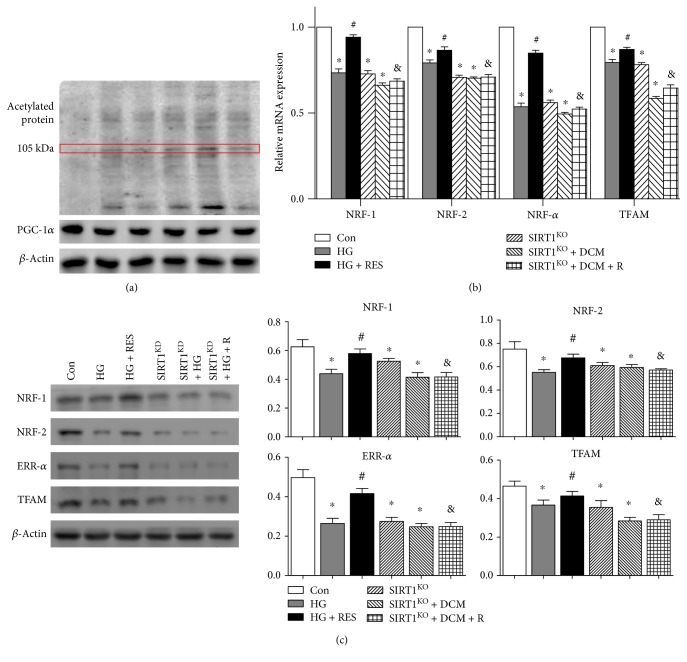
SIRT1 regulated mitochondrial-related proteins expression through PGC-1*α* deacetylation. (a) Nonfunctional PGC-1*α* acetylation (about 105 kDa, indicated by the red box) was upregulated in the HG and SIRT1^KD^ groups; meanwhile, SIRT1 activation in HG + RES cells promoted functional PGC-1*α* deacetylation. (b) HG and SIRT1^KD^ decreased the mRNA expression of mitochondrion-related genes such as *NRF-1*, *NRF-2*, *ERR*-*α*, and *TFAM* (^∗^*p* < 0.05). SIRT1 activation by resveratrol upregulated the expression of *NRF-1*, *NRF-2*, *ERR-α*, and *TFAM* mRNA expression in HG + RES cells but not in SIRT1^KD^ + HG + R cells (^#^*p* < 0.05, ^&^*p* < 0.05). (c) Protein expression level changes of NRF-1, NRF-2, ERR-*α*, and TFAM showed the similar tendency with mRNA. ^∗^*p* < 0.05 versus Con; ^#^*p* < 0.05 versus DCM; ^&^*p* < 0.05 versus DCM + RES. NRF-1: nuclear respiratory factor 1; NRF-2: nuclear respiratory factor 2; ERR-*α*: estrogen-related receptor-*α*; TFAM: mitochondrial transcription factor A.

To demonstrate the signaling pathway of SIRT1 in mitochondrial regulation, we detected acetylated protein in the six groups to reveal the level of acetylated PGC-1*α* (about 105 kDa, indicated by the red box). Since SIRT1 expression was reduced in HG, SIRT1^KD^, and SIRT1^KD^ + HG + R cells, nonfunctional PGC-1*α* acetylation was upregulated in the HG and SIRT1^KD^ groups; meanwhile, SIRT1 activation in HG + RES cells promoted functional PGC-1*α* deacetylation (Figure 5(a)). As indicated in Figure 5(b), HG and SIRT1^KD^ decreased the mRNA expression of mitochondrion-related genes such as nuclear respiratory factor 1 (NRF-1), nuclear respiratory factor 2 (NRF-2), estrogen-related receptor-*α* (ERR-*α*), and mitochondrial transcription factor A (TFAM), and SIRT1 activation by resveratrol upregulated the expression of *NRF-1*, *NRF-2*, *ERR-α*, and *TFAM* mRNA expression in HG + RES cells but not in SIRT1^KD^ + HG + R cells (*NRF-1*: 0.73 ± 0.02/Con, *p* < 0.05, HG versus Con; 0.73 ± 0.02/Con, *p* < 0.05, SIRT1^KD^ versus Con; 0.94 ± 0.01 versus 0.73 ± 0.02, *p* < 0.05, HG + RES versus HG; 0.69 ± 0.01 versus 0.66 ± 0.01, *p* > 0.05, SIRT1^KD^ + HG + RES versus SIRT1^KD^ + HG; *NRF-2*: 0.79 ± 0.02/Con, *p* < 0.05, HG versus Con; 0.71 ± 0.01/Con, *p* < 0.05, SIRT1^KD^ versus Con; 0.87 ± 0.02 versus 0.79 ± 0.02, *p* < 0.05, HG + RES versus HG; 0.71 ± 0.02 versus 0.70 ± 0.01, *p* > 0.05, SIRT1^KD^ + HG + RES versus SIRT1^KD^ + HG; *ERR-α*: 0.54 ± 0.02/Con, *p* < 0.05, HG versus Con; 0.56 ± 0.02/Con, *p* < 0.05, SIRT1^KD^ versus Con; 0.85 ± 0.02 versus 0.54 ± 0.02, *p* < 0.05, HG + RES versus HG; 0.52 ± 0.01 versus 0.49 ± 0.01, *p* > 0.05, SIRT1^KD^ + HG + RES versus SIRT1^KD^ + HG; and *TFAM*: 0.79 ± 0.02/Con, *p* < 0.05, HG versus Con; 0.78 ± 0.01/Con, *p* < 0.05, SIRT1^KD^ versus Con; 0.87 ± 0.01 versus 0.79 ± 0.02, *p* < 0.05, HG + RES versus HG; 0.65 ± 0.02 versus 0.59 ± 0.01, *p* > 0.05, SIRT1^KD^ + HG + RES versus SIRT1^KD^ + HG).  Figure 5(c) reveals expression level changes of mitochondrion-related downstream proteins of NRF-1, NRF-2, ERR-*α*, and TFAM. Protein expressions of NRF-1, NRF-2, ERR-*α*, and TFAM were decreased in HG and decreased in SIRT1^KD^; meanwhile, SIRT1 activation by resveratrol upregulated the expression of NRF-1, NRF-2, ERR-*α*, and TFAM expressions in HG + RES cells but not in SIRT1^KD^ + HG + R cells (NRF-1: 0.44 ± 0.05 versus 0.63 ± 0.09, *p* < 0.05, HG versus Con; 0.53 ± 0.03 versus 0.63 ± 0.09, *p* < 0.05, SIRT1^KD^ versus Con; 0.58 ± 0.06 versus 0.44 ± 0.05, *p* < 0.05, HG + RES versus HG; 0.42 ± 0.05 versus 0.41 ± 0.06, *p* > 0.05, SIRT1^KD^ + HG + RES versus SIRT1^KD^ + HG; NRF-2: 0.55 ± 0.04 versus 0.75 ± 0.11, *p* < 0.05, HG versus Con; 0.61 ± 0.05 versus 0.75 ± 0.11, *p* < 0.05, SIRT1^KD^ versus Con; 0.68 ± 0.06 versus 0.55 ± 0.04, *p* < 0.05, HG + RES versus HG; 0.57 ± 0.02 versus 0.59 ± 0.04, *p* > 0.05, SIRT1^KD^ + HG + RES versus SIRT1^KD^ + HG; ERR-*α*: 0.26 ± 0.05 versus 0.50 ± 0.07, *p* < 0.05, HG versus Con; 0.27 ± 0.04 versus 0.50 ± 0.07, *p* < 0.05, SIRT1^KD^ versus Con; 0.42 ± 0.05 versus 0.26 ± 0.05, *p* < 0.05, HG + RES versus HG; 0.25 ± 0.04 versus 0.25 ± 0.03, *p* > 0.05, SIRT1^KD^ + HG + RES versus SIRT1^KD^ + HG; and TFAM: 0.37 ± 0.05 versus 0.46 ± 0.05, *p* < 0.05, HG versus Con; 0.35 ± 0.06 versus 0.46 ± 0.05, *p* < 0.05, SIRT1^KD^ versus Con; 0.41 ± 0.05 versus 0.37 ± 0.05, *p* < 0.05, HG + RES versus HG; 0.29 ± 0.05 versus 0.28 ± 0.03, *p* > 0.05, SIRT1^KD^ + HG + RES versus SIRT1^KD^ + HG).

## 4. Discussion

Diabetic cardiomyopathy (DCM) is one of the most important causes for morbidity and mortality in diabetic patients, characterized by diastolic dysfunction in early stages, proceeding decreased systolic function and eventual heart failure, which are independent of other cardiac diseases such as coronary heart disease or atherosclerosis [25]. The pathogenesis of DCM is not clearly illuminated, and existing treatment options are limited. Several studies considered mitochondria as a promising target for the management of DCM. Our present study demonstrated that cardiac-specific knockdown of SIRT1 is sufficient to cause phenotypes resembling DCM in mice heart and that SIRT1 played a beneficial role against the development of DCM. SIRT1 activation by resveratrol alleviated decreased cardiac function, impaired mitochondrial biogenesis, and function in DCM mice. Furthermore, SIRT1 improved mitochondrial dynamics through the deacetylation of PGC-1*α* and regulation of downstream proteins such as NRF-1, NRF-2, ERR-*α*, and TFAM.

Clinically, diabetes mellitus (DM) is categorized into type 1 DM and type 2 DM [26, 27]. Type 1 insulin-dependent DM accounts for about ten percent while type 2 DM is considered as the etiology of over 80 percent of all diabetics. Apart from a single state of hyperglycemia, type 2 diabetes is usually accompanied by obesity-induced insulin resistance and hyperinsulinaemia, which could have a nonnegligible effect on insulin signaling and lead to cardiac hypertrophy [28]. The use of type 1 diabetic model could effectively avoid these changes. In most animal studies of type 1 diabetes mellitus, diabetes is induced by the administration of pancreatic beta-cell toxin streptozotocin (STZ) [29]. *In vivo* researches in these rodent models have provided echocardiography evidence for systolic and diastolic dysfunction [30, 31]. Some studies were also performed in isolated perfused hearts and revealed depressed cardiac function, according to a recent review by Severson [32]. Additionally, animal models provide the opportunity to conduct mechanistic studies for DCM. Several hypotheses have been proposed, including impaired calcium homeostasis, activation of the renin-angiotensin system, increased oxidative stress, mitochondrial dysfunction, and altered substrate metabolism [29]. In our present study, we established type 1 diabetic model by STZ administration even though the fact is that type 2 diabetes is more popular than type 1 diabetes in humans. Cardiac hypertrophy and fibrosis are two essential characteristics of DCM that caused diabetic cardiac dysfunction. As shown in our Results, STZ-induced DCM model was accompanied by enhanced ventricle weight, increased ANP and BNP level, myocardial fibrosis, and impaired cardiac function.

Considering the multiple functions of SIRT1 in various cell types, generalized knockout of SIRT1 may exert a complicated effect on metabolism in the whole body, which would confuse the results in our study. Furthermore, high perinatal mortality was reported in generalized SIRT1-deficient mice [33], and whole body SIRT1 knockouts suffer from severe growth retardation and a number of developmental defects [34]. Thus, we used Cre-loxP recombination system, by which the desired gene modification can be restricted to certain cell types, to generate cardiac-specific SIRT1 knockout mice to avoid above-mentioned conditions. [35]. As shown in our Western blot results, SIRT1 protein was specifically knocked out in heart tissue while normally expressed in other tissues such as lung, brain, and kidney. Additionally, specific SIRT1 deletion in myocardium did not affect the survival rate and whole body weight in SIRT1^KO^ mice, which indicated that SIRT1 deficiency did not affect growth and development in immature mice. A major innovative finding of our present study is that cardiac-specific knockdown of SIRT1 is sufficient to induce cardiac phenotypes resembling DCM in mice. Cardiac function in SIRT1^KO^ mice was markedly reduced as compared with that in WT mice, accompanied with cardiac hypertrophy and fibrosis, indicating the crucial role of SIRT1 in cardiac function. Moreover, interestingly, cardiac function reduction in DCM mice was accompanied by decreased SIRT1 expression, demonstrating that the impaired heart function was associated with SIRT1 deficiency.

Even though antidiabetic effects of resveratrol have been widely studied, the low bioavailability of resveratrol raises questions about whether the beneficial effects of oral resveratrol can act directly on diabetic myocardial tissue [22]. We show here that intraperitoneal injection of resveratrol reversed DCM-induced reduction in SIRT1 protein level while also enhancing cardiac function in DCM heart. However, due to the multifunctional properties of resveratrol, it could also exert beneficial effects against DCM via other mechanisms, such as antioxidant and anti-inflammatory effects [36]. A study by Guo et al. demonstrated that resveratrol attenuated high glucose-induced oxidative stress and cardiomyocyte apoptosis through the suppression of NADPH oxidase-derived ROS generation and the activation of antioxidant defenses via the regulation of AMPK pathway [37]. Additionally, Huang et al. reported that resveratrol prevented cardiac dysfunction in diabetes by relieving nitrosative and oxidative stress [38]. In a recent study by Bagul and his colleagues, it was revealed that resveratrol ameliorated cardiac oxidative stress in diabetes through deacetylation of NF-kB and histone 3 [39]. In this current study, cardiac-specific SIRT1 knockout mice provided the opportunity to directly assess the effects of resveratrol in animals lacking functional SIRT1. Using this model, we clearly demonstrate that the ability of resveratrol to improve cardiac function in DCM is, at least partially, dependent on SIRT1.

Currently, the underlying mechanisms for the physiopathologic progression of DCM remain exclusive. Abundant evidence has shown that DCM is associated with multiple factors including impaired myocardial insulin signaling and calcium homeostasis, mitochondrial dysfunction, endoplasmic reticulum stress, abnormal coronary microcirculation, and activation of the sympathetic nervous system or renin-angiotensin-aldosterone system. These changes lead to excessive oxidative stress, myocardial fibrosis, cardiac diastolic dysfunction, and eventually systolic heart failure [40]. DCM-associated myocardial apoptosis and fibrosis contributed to the loss of cardiac function [41]. In accordance with previous findings, we found enhanced apoptosis and fibrosis in diabetic hearts accompanied by a significant reduction in cardiac function of EF and FS. Interestingly, similar tendency was found in SIRT1^KO^ mice. SIRT1 activation by resveratrol markedly reversed these changes in DCM mice but not in DCM + SIRT1^KO^ mice. Our results manifested that SIRT1 played an essential role in myocardium, and downregulation of SIRT1 due to DCM or SIRT1^KO^ was associated with cardiac dysfunction and increase in myocardial apoptosis and fibrosis.

It is well recognized that in diabetic hearts, the use of glucose is decreased. As evidenced by our data, SIRT1 is essentially involved in the regulation of cardiac metabolism. Similar to DCM hearts, the glucose uptake is inhibited in SIRT1 low-expressed hearts. More importantly, we provided evidence that SIRT1 knockdown led to cardiac insulin resistance, as low expression of SIRT1 caused reduced IRS2 expression and impaired insulin signaling in both *in vivo* and *in vitro* models. Insulin resistance is associated with mitochondrial dysfunction due to reduced insulin-stimulated mitochondrial activity [42].

Mitochondria are the center of cellular metabolism and, thus, are highly linked to impaired metabolism associated with DCM. Significant data indicates that mitochondrion alterations may play an essential role in the development of DCM [43]. Here, our results demonstrate that a normal heart showed regular tightly packed interfibrillar mitochondria while DCM and SIRT1^KO^ hearts displayed swollen mitochondria with loss of discernable cristae and accumulation of glucose granules [44]. It was also reported that STZ-treated mice had significant changes to interfibrillar mitochondrial population, including reduced mitochondrial size, cardio-lipid content, and electron transport activity [45]. Apart from morphological mitochondrial abnormities, the reduction of mitochondrial DNA (mtDNA) amount both in *in vivo* and *in vitro* under HG and SIRT1^KO^ conditions suggested a dysregulation in mitochondrial biogenesis. We further investigate mitochondrial functional indexes including mitochondrial complex IV enzyme activity and ATP production. Myocardium has a high rate of ATP production, and turnover is required to maintain continuous mechanical work. Normal myocardium depends on abundant mitochondrial ATP supply to properly develop force. During DCM, however, myocardial energetic balance is disturbed, contributing to the systolic and diastolic dysfunction. However, it must be noted that even though we observed concurrent mitochondrial dysfunction and altered mitochondrial morphology in DCM myocardium, the causative nature between functional and morphological changes of mitochondria in DCM is unclear [46]. In a recent study by Ni et al., mitochondrial ATP synthase and insufficient ATP production are important mechanisms contributing to DCM [47]. Their results are partially in accordance with our present, but they focus on ATP synthase complex V. In our current study, we found decreased complex IV enzyme activity and reduced ATP generation in HG and SIRT1^KO^ cells. Resveratrol increased complex IV enzyme activity and ATP production under HG condition, but this effect was diminished when SIRT1 was deficient. Mitochondrial dysfunction and abnormal biogenesis are central upstream defect inflicted on the heart of DCM [48]; herein, we demonstrate the possibility of targeting mitochondrial energetics through the activation of SIRT1 for the management of DCM.

As a result of these findings above, there has been a surge of interest in understanding the molecular mechanism and targets of SIRT1's protective effect against DCM. Notably, one gene whose decreased expression is consistently implicated in diabetic mice is the peroxisome proliferator-activated receptor *γ* coactivator (PGC-1*α*) [49–51]. PGC-1*α*, a transcriptional coactivator, plays a central role in the regulation of myocardial metabolism and mitochondrial biogenesis. Although PGC-1*α* was reported to be upregulated in diabetes, we found unchanged PGC-1*α* expression in DCM and SIRT1^KO^ hearts. However, acetylated level of PGC-1*α* was altered, contributing to functional PGC-1*α* changes. SIRT1 physically interacted with deacetylated PGC-1*α*, consequently increasing PGC-1*α* activity. As a consequence, functional deacetylated PGC-1*α* was decreased under the condition of DCM and SIRT1^KO^, leading to decreased mRNA and protein expression of mitochondrion-related genes of NRF-1, NRF-2, ERR-*α*, and TFAM. NRF-1, NRF-2, ERR-*α*, and TFAM are four of the most essential mitochondrial regulatory genes. The regulation effect of NRF-1 and NRF-2 on nucleus-encoded mitochondrial transcription factors is essential to the control of mitochondrial biogenesis [52]. Functional PGC-1 by SIRT1 stimulated a powerful induction of NRF-1 and NRF-2 gene expression, consequently increasing the expression of proteins involved in oxidative phosphorylation, and thus played an important role in the regulation of mitochondrial biogenesis and function [53]. ERR-*α* responds to signals central to the regulation of mitochondrial biogenesis and function, such as upon exposure to cold, fasting, and exercise [54, 55]. Previous studies have shown that ERR-*α* is required for the ability of exogenously expressed PGC-1*α* to induce mitochondrial biogenesis and respiration [56]. Of particular, TFAM is a main regulator for the mtDNA copy number and plays a critical role in the stability of mtDNA via formation of nucleoid structure. TFAM is a key factor for mtDNA maintenance, and the expression of human TFAM in a mouse increased the amount of mtDNA almost in parallel with the increase in the TFAM [57].

However, there are some limitations in our current study. Firstly, even through resveratrol is widely used as an activator for SIRT1, its agonist effect is not exclusive to SIRT1. Chen et al. reported the activation effect of resveratrol on SIRT3 and the consequential cardiac protective effect [58]. Although we used SIRT1^KO^ transgenic mice and lentiviral vector targeting SIRT1 shRNA to testify that the beneficial effect of resveratrol was dependent on SIRT1 activation, specific SIRT1 agonist is still needed to obtain more convincing results. Secondly, as SIRT1 can interact with a variety of mitochondrion-related proteins apart from PGC-1*α*, more molecular experiments are needed to investigate more about the signaling pathway.

## 5. Conclusion

In conclusion, we have demonstrated that the expression of SIRT1 was markedly reduced in DCM hearts. And we have found, for the first time, that cardiac-specific low expression of SIRT1 caused both compromised insulin signaling and mitochondrial dynamic abnormity, contributing to phenotypes resembling DCM in the mouse heart. Our results strongly support the conclusion that SIRT1 have a beneficial effect on cardiac dysfunction caused by DCM or HG through mitochondrial biogenesis and functional regulations. Furthermore, the protective role of SIRT1 on mitochondria is via the deacetylation effect on PGC-1*α*, thus inducing the increased expression of mitochondrial regulatory genes of NRF-1, NRF-2, ERR-*α*, and TFAM. Collectively, SIRT1 may serve as a potential therapeutic target for the management of DCM.

## Supplementary Material

Fig. S1 Normal SIRT1 expression in other tissues in SIRT1KO mice. There was no significant difference in SIRT1 mRNA (A) and protein (B) expressions in other organs such as lung, kidney and brain in WT, Heter or SIRT1KO mice. Fig. S2 SIRT1 down-regulation in H9c2 cells by shRNA lentiviral vector (A) GFP fluorescence images and flow cytometry results (B) showed that H9c2 cells were optimally transfected at the MOI of 100. (C) No. 1 sequence shRNA decreased SIRT1 expression to the largest extent (∗∗p < 0.01). (D) HG treatment significantly reduced SIRT1 expression in H9c2 cells (∗p < 0.05), and resveratrol pointedly elevated SIRT1 in HG cells (#p < 0.05). Additionally, resveratrol did not reverse SIRT1 down-regulation in SIRT1KD cells due to the knock-down efficacy of shRNA (&p < 0.05). ∗p < 0.05 vs. Con; ∗∗p < 0.01 vs. Con; #p < 0.05 vs. DCM; &p < 0.05 vs. DCM + RES.

## Figures and Tables

**Table 1:** Primer sequences for real-time PCR.

	Forward primer	Reverse primer
*SIRT1*	ACAACCTCCTGTTGGCTGATG	GCTTGCGTGTGATGCTCTGT
*NRF-1*	GCACCTTTGGAGAATGTGGT	GGGTCATTTTGTCCACAGAGA
*NRF-2*	CCAGCTACTCCCAGGTTGC	CCTGATGAGGGGCAGTGA
*TFAM*	CCTTCGATTTTCCACAGAACA	GCTCACAGCTTCTTTGTATGCTT
*ERR-α*	CAAGAGCATCCCAGGCTT	GCACTTCCATCCACACACTC
*ANP*	GCTTCCAGGCCATATTGGAGCA	TCTCTCAGAGGTGGGTTGACCT
*BNP*	ATGGATCTCCTGAAGGTGCTGT	GCAGCTTGAGATATGTGTCACC
*GAPDH*	GGCACAGTCAAGGCTGAGAATG	ATGGTGGTGAAGACGCCAGTA
