# Expression of Concern: “SIRT1 Activation by Resveratrol Alleviates Cardiac Dysfunction via Mitochondrial Regulation in Diabetic Cardiomyopathy Mice”

**DOI:** 10.1155/omcl/9834761

**Published:** 2026-09-28

EXPRESSION OF CONCERN: S. Ma, J. Feng, R. Zhang, et al., “SIRT1 Activation by Resveratrol Alleviates Cardiac Dysfunction via Mitochondrial Regulation in Diabetic Cardiomyopathy Mice,” *Oxidative Medicine and Cellular Longevity*, vol. 2017 (2017). https://doi.org/10.1155/2017/4602715.

This Expression of Concern is for the above article, published online on 13 August 2017 in Wiley Online Library (wileyonlinelibrary.com), and has been issued following the agreement of the Journal’s Editor‐in‐Chief, Jeannette Vasquez‐Vivar; and John Wiley & Sons Ltd.

The Expression of Concern has been issued due to the investigation of concerns flagged by a third party on PubPeer [1]. The concerns relate to the similarity of the Western Blot bands corresponding to “P‐AktS473” expression in Figure 3e.

The journal could not obtain the raw data from the authors due to the article’s age and could not definitively establish that the above‐mentioned concerns affect the overall conclusions of the article. Therefore, the journal has decided to issue an Expression of Concern to inform and alert readers. The authors have been notified.
