# Supplementary material for: SIRT1 Activation by Resveratrol Alleviates Cardiac Dysfunction via Mitochondrial Regulation in Diabetic Cardiomyopathy Mice
Source: Oxid Med Cell Longev. 2017 Aug 13;2017:4602715. doi: 10.1155/2017/4602715 (PMC5572590; doi:10.1155/2017/4602715)
Supplement: Supplementary file 1 — Fig. S1 Normal SIRT1 expression in other tissues in SIRT1KO mice. There was no significant difference in SIRT1 mRNA (A) and protein (B) expressions in other organs such as lung, kidney and brain in WT, Heter or SIRT1KO mice. Fig. S2 SIRT1 down-regulation in H9c2 cells by shRNA lentiviral vector (A) GFP fluorescence images and flow cytometry results (B) showed that H9c2 cells were optimally transfected at the MOI of 100. (C) No. 1 sequence shRNA decreased SIRT1 expression to the largest extent (∗∗p < 0.01). (D) HG treatment significantly reduced SIRT1 expression in H9c2 cells (∗p < 0.05), and resveratrol pointedly elevated SIRT1 in HG cells (#p < 0.05). Additionally, resveratrol did not reverse SIRT1 down-regulation in SIRT1KD cells due to the knock-down efficacy of shRNA (&p < 0.05). ∗p < 0.05 vs. Con; ∗∗p < 0.01 vs. Con; #p < 0.05 vs. DCM; &p < 0.05 vs. DCM + RES. [file 4602715.f1.docx]

**Supplementary materials**


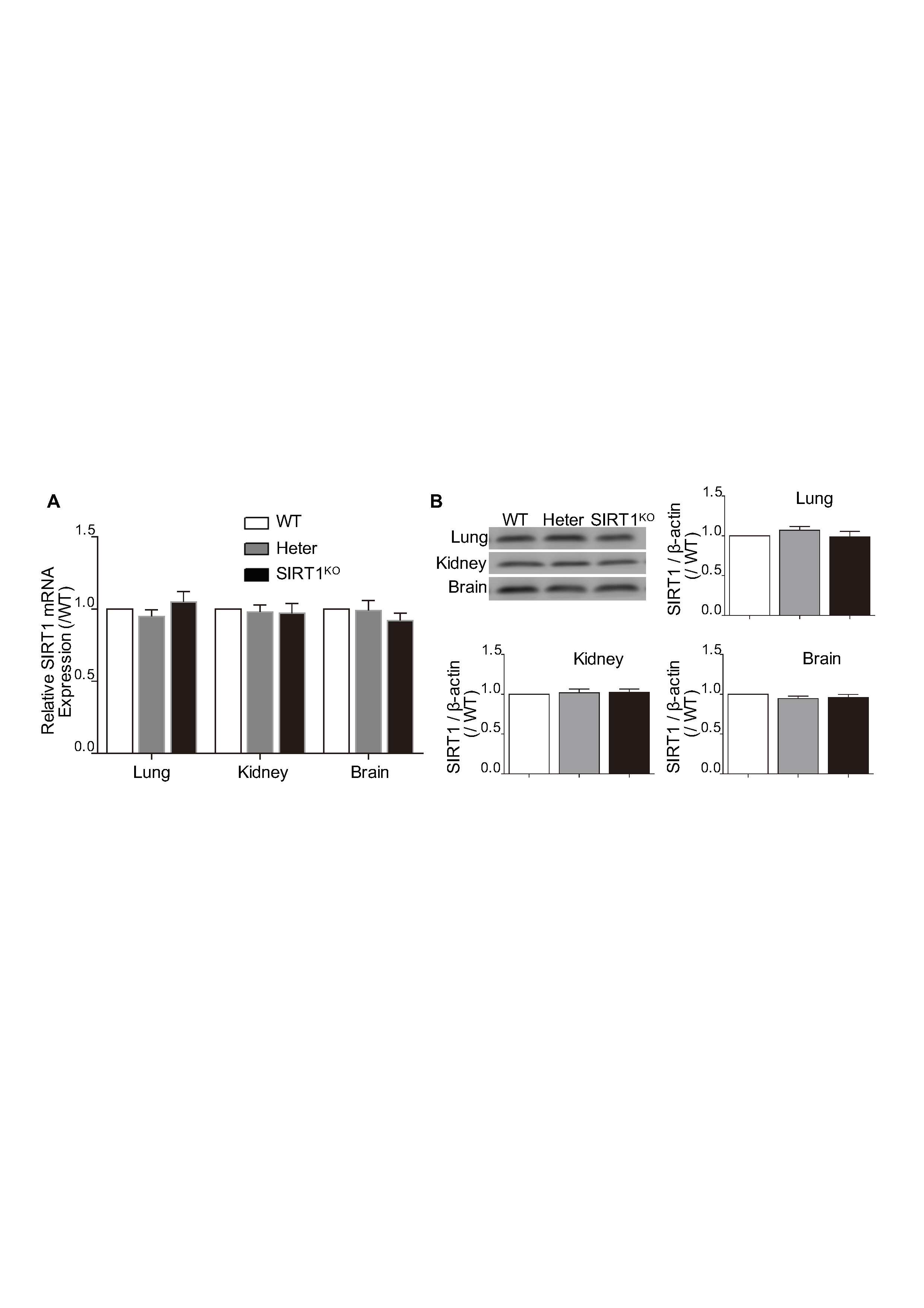


**Fig. S1 Normal SIRT1 expression in other tissues in SIRT1^KO^ mice**

There was no significant difference in SIRT1 mRNA (A) and protein (B) expressions in other organs such as lung, kidney and brain in WT, Heter or SIRT1^KO^ mice.

**
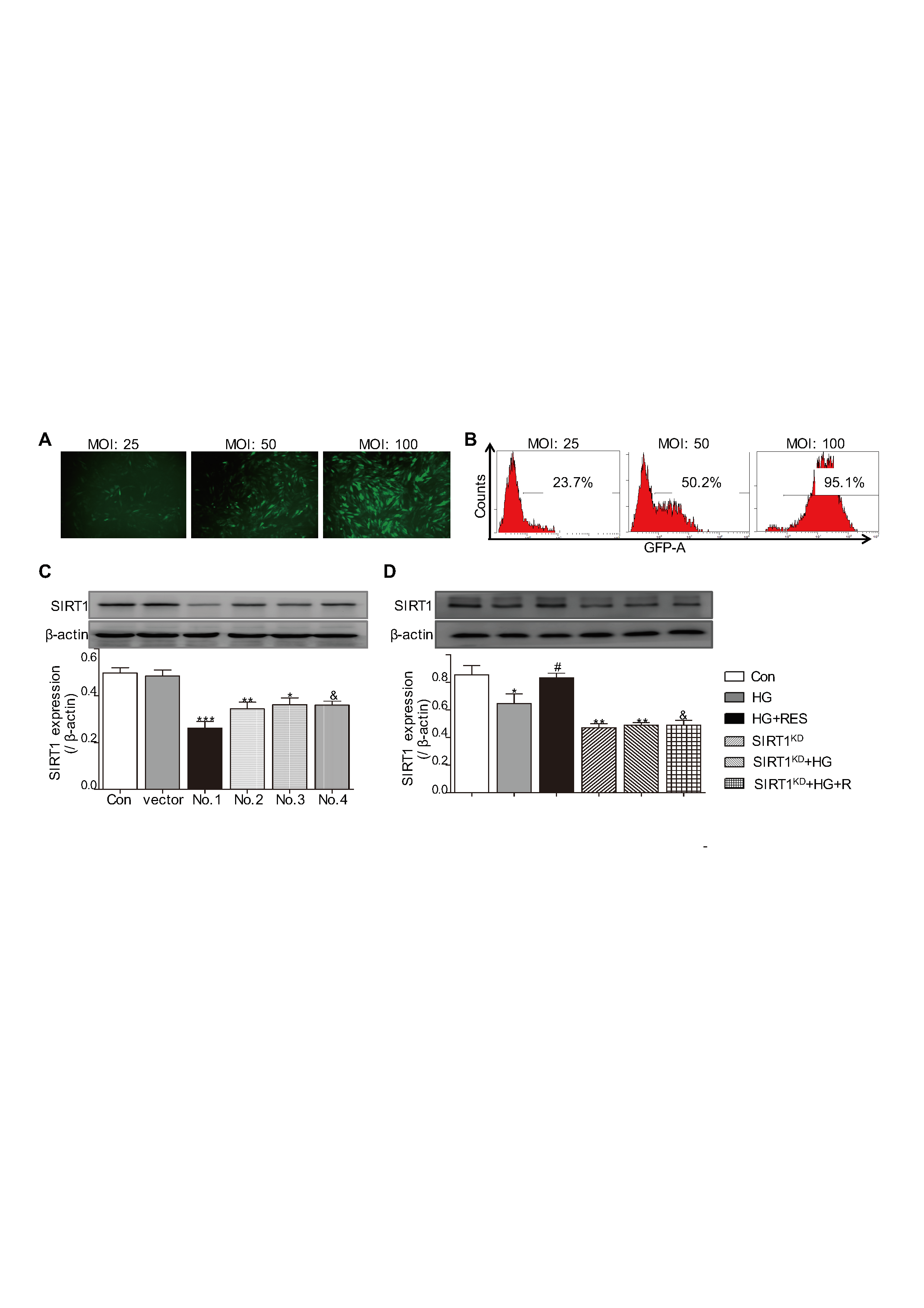
**

**Fig. S2 SIRT1 down-regulation in H9c2 cells by shRNA lentiviral vector**

(A) GFP fluorescence images and flow cytometry results (B) showed that H9c2 cells were optimally transfected at the MOI of 100. (C) No. 1 sequence shRNA decreased SIRT1 expression to the largest extent (***p* < 0.01). (D) HG treatment significantly reduced SIRT1 expression in H9c2 cells (**p* < 0.05), and resveratrol pointedly elevated SIRT1 in HG cells (^#^*p* < 0.05). Additionally, resveratrol did not reverse SIRT1 down-regulation in SIRT1^KD^ cells due to the knock-down efficacy of shRNA (^&^*p* < 0.05).

**p* < 0.05 vs. Con; ***p* < 0.01 vs. Con; ^#^*p* < 0.05 vs. DCM; ^&^*p* < 0.05 *vs.* DCM + RES
